# The recurrence risk of neural tube defects (NTDs) in a population with high prevalence of NTDs in northern China

**DOI:** 10.18632/oncotarget.19890

**Published:** 2017-08-03

**Authors:** Jufen Liu, Zhiwen Li, Nicholas D.E. Greene, Hongtian Li, Aiguo Ren

**Affiliations:** ^1^ Institute of Reproductive and Child Health/Key Laboratory of Reproductive Health, National Health and Family Planning Commission of the People's Republic of China, Peking University, Beijing, P. R. China; ^2^ Department of Epidemiology and Biostatistics, School of Public Health, Peking University, Beijing, P. R. China; ^3^ UCL Great Ormond Street Institute of Child Health, University College London, London, United Kingdom

**Keywords:** recurrence, neural tube defects, folic acid, supplementation, China

## Abstract

**Background:**

Although a number of studies have reported the recurrence risk of NTDs in developed countries, there is little data on the rate of recurrence of NTDs in northern China, a region of high prevalence of NTDs.

**Methods:**

Based on the population-based birth defects surveillance system of five counties, we identified women who had an NTD affected pregnancy from 2004-2015 and a retrospective survey was conducted. The rate of recurrence of NTDs was calculated by the number of recurrent NTDs divided by the first NTDs. Maternal age, body mass index (BMI), gestational weeks, education, and occupation were collected. Information on folic acid (FA) supplements, time and dosage were also recorded.

**Results:**

Among 851 women who had a previous NTD-affected pregnancy, there were 578 subsequent pregnancies, with 10 recurrent NTDs, a 1.7% recurrence rate. The recurrence rate was 1.5% and 2.6% for those taking FA supplements and without FA supplementation respectively. Women with recurrent NTDs had higher BMI before pregnancy compared to those who had a second pregnancy without NTDs. Among the recurrent NTDs, the majority were spina bifida.

**Conclusions:**

The recurrence rate of NTDs was approximately five times higher than the overall prevalence in the same region of northern China. Risk of recurrence appeared lower among women who took FA supplements. These findings are consistent with the reduction in NTD frequency in the population since introduction of the nationwide FA supplement program. Data on recurrence rates in northern China will inform power calculations for future intervention studies.

## INTRODUCTION

Neural tube defects (NTDs), such as anencephaly and spina bifida are common and severe malformations of the developing central nervous system. Women who have had a pregnancy affected by an NTD have an elevated risk of a subsequent NTD-affected pregnancy [1]. Several studies report the recurrence risk of NTDs in developed countries [2–4]. The recurrence rate was 3.0% (95% confidence limits: 2.0-4.3%) in the United States in the late 1970s [5], 2.9% in Belgium [4] and 1.2-1.7% in England [2, 3]. However, these studies have limited sample size, typically less than around one or two hundred [2–4], with only one study including around 1000 cases [6].

The prevalence of NTDs in China is among the highest worldwide [7], but studies reporting prevalence have not categorized the risk of occurrence and recurrence [8–10]. For example, the prevalence of total NTDs was 1.2% in 2004 and 0.79% in 2009, declining to 0.32% in 2014 [10]. Hence, prevalence is approximately 10 times higher than rates in the USA and Europe [11, 12]. Only one study reported an estimate of recurrence risk of NTDs, based on a retrospective survey in the early 1990s. The overall recurrence was 6.9%, with differing rates in low, average and high prevalence areas of 3.7%, 4.5% and 8.3%, respectively [13]. Because the recurrence rate of NTDs was related to the incidence of NTDs in the general population [5, 14], and the prevalence of NTDs has declined dramatically in recent years, the recurrence risk of NTDs may be quite different from two decades ago, in particular since the introduction of a folic acid supplementation programme.

Maternal supplementation with folic acid (FA) in the periconceptional period significantly reduces the risk of NTDs, both occurrent and recurrent [6, 15–17]. A meta-analysis of the randomized trials indicated a 69%-87% reduction with use of FA for the prevention of recurrent NTDs and a 85–100% reduction in observational studies [1]. However, some of those studies were conducted prior to the widespread use of folic acid supplements and fortification of enriched cereal grain products [5], and some compared a specific vitamin supplement, for example, Pregnavite Forte F [2]. The prevention effect of population-based supplementation of folic acid has not been studied in Chinese people.

Using a population-based birth-defect surveillance system and a retrospective survey, the current study aimed to examine the recurrence of NTDs in 5 counties of Shanxi province, China, and to gain information on the impact of the FA supplement program since 2009. The study addressed three main issues, with the major aim being to determine the risk of NTD recurrence in a population with a high prevalence of NTDs. Our second aim was to evaluate, in a population with a first affected pregnancy, the use of FA supplements in a subsequent pregnancy, to determine whether FA supplementation had any protective effects. The third aim was to obtain information on the dietary characteristics of women who suffer recurrent NTDs.

## RESULTS

In all, 1547 NTD cases were identified from 2004 to 2015 in the five counties, and 851 cases were followed up in this survey, a follow-up rate of 55%. There were no apparent significant differences between mothers who participated in the follow-up study and cases in which the mother did not participate. There were 578 subsequent pregnancies following 851 first NTDs, with 10 recurrent NTDs, corresponding to an overall recurrence rate of 1.7% (10/578). The recurrence rate was 1.5% (7/456) in those taking FA supplements and 2.6% (3/117) in those who were not taking such supplements ( *P*=0.434).

There were no significant differences in age, occupation, or education between women with recurrent NTD pregnancies and those with unaffected pregnancies. However, women who suffered a recurrent NTD had a higher BMI before pregnancy than those who did not (*P*<0.05) (Table 1).

**Table 1:** Characteristics of the subjects [M (SD) or n (%)]

	Characteristics	Women with first NTDs (N=851)	Women with second pregnancy after occurrent NTD (N=578)	Women with recurrent NTDs (N=10)	Women without recurrent NTDs (N=568)	P^*^
	**Age**	28.0(5.6)	26.5(4.8)	26.4(4.6)	26.5(4.8)	0.955
	**BMI**	23.5(3.3)	23.4(3.2)	27.5(5.2)	23.3(3.2)	<0.001
	**Gestational weeks**	25.3(8.0)	24.7(7.5)	22.1(4.5)	39.2(3.5)	<0.001
**Occupation**	Farmers	636(74.7)	423(73.2)	8(80.0)	415(73.1)	0.855
	Others	205(24.1)	148(25.6)	2(20.0)	146(25.7)	
	Missing	10(1.2)	7(1.2)	0	7(1.2)	
**Education**	Primary school	104(12.2)	60(10.4)	2(20.0)	58(10.2)	0.754
	Junior high school	613(72.0)	423(73.2)	7(70.0)	416(73.2)	
	Senior high school and above	126(14.8)	92(15.9)	1(10.0)	91(16.0)	
	Missing	8(0.9)	3(0.5)	0	3(0.5)	

^*^ Compared between recurrent NTDs and the second pregnancy without NTDs

The proportion of women who took FA supplements in their second pregnancy was lower among women who suffered recurrent NTDs than those who did not (*P*<0.05) (Table 2). Among the first NTD pregnancies, 73% of women reported taking FA supplements after they knew they were pregnant which was too late to prevent NTDs. In a recurrent NTD, 30% of women took FA after pregnancy (Table 3). The majority of women in each group took FA at a dose of 0.4 mg per day and only one individual took more than 5 mg per day.

**Table 2:** Characteristics of NTDs in first and recurrent pregnancy [n(%)]

	Characteristics	First NTDs (N=851)	Second pregnancy (N=578)	Recurrent NTDs (N=10)	Second pregnancy without NTDs (N=568)	P^*^
Types of defects						
	Anencephaly	379(44.5)	3(0.5)	3(30.0)		
	Spina bifida	398(46.8)	6(1.0)	6(60.0)		
	Encephalocele	74(8)	1(0.2)	1(10.0)		
	Other type		19(3.3)	0		
	No BD		542(93.8)			
	Male	383(45.0)	309(53.5)	5(50.0)	304(53.5)	<0.001
Sex	Female	372(43.7)	258(44.6)	3(30.0)	255(44.9)	
	Bisexual	1(0.1)	0			
	Unknown	95(11.2)	11(1.9)	2(20.0)	9(1.6)	
FA supplements						
	Yes	233(27.4)	455(78.7)	7(70.0)	449(79.0)	0.012
	No	586(68.9)	117(20.2)	3(30.0)	114(20.1)	
	Missing	32(3.8)	6(1.0)		5(0.9)	
FA supplements before pregnancy						
	Yes	63(7.4)	276(47.8)	3(30.0)	273(48.1)^*^	

^*^ Compared between recurrent NTDs and the second pregnancy without NTDs

**Table 3:** Description of folic acid supplement for the first and second pregnancy (among those taking folic acid)

Folic acid (FA) supplement	First NTDs (N=233)	Second pregnancy (N=455)	Recurrent NTDs (N=7)	Second pregnancy without NTDs (N=449)	P^*^
Period of FA supplementation					0.770
3 months before pregnancy	31(13.3)	218(37.7)	3(43.0)	215(37.9)	
2 months before pregnancy	7(3.0)	27(4.7)	0	27(4.8)	
1 month before pregnancy	25(10.7)	31(5.4)	0	31(5.5)	
After pregnancy	170(73.0)	179(31.0)	4(57.0)	175(30.8)	
Missing	0	123(21.3)	0	120(21.1)	
FA supplements dose					0.722
0.4 mg		331(57.3)	4(57.0)	327(57.6)	
0.8 mg		36(6.2)	1(14.0)	35(6.2)	
1-3 mg		6(1.0)	0	6(1.1)	
≥4 mg		69(11.9)	1(14.0)	68(12.0)	
Missing		136(23.5)	1(14.0)	132(23.2)	

^*^ Compared between recurrent NTDs and the second pregnancy without NTDs

The majority of first NTDs were anencephaly or spina bifida, which accounted for 80% of all NTDs. The types of NTDs affecting successive pregnancies for each mother were not necessarily the same in each case. Three pregnancies affected by anencephaly were followed by spina bifida in the second pregnancy, two spina bifida-affected pregnancies were followed by anencephaly, and one anencephaly was followed by an encephalocele (Table 4). Male and female babies were both represented among recurrent NTDs. In addition to NTDs, a number of other malformations or abnormal post-natal outcomes were also reported among women who had previously suffered an NTD-affected pregnancy (Table 4).

**Table 4:** Characteristics of two pregnancies with birth defects by types [n(%)]

First pregnancy	Second pregnancy	Frequency
Anencephaly	Spina bifida	3
Anencephaly	Cleft palate	1
Anencephaly	Encephalocele	1
Anencephaly	Miscarriage	1
Anencephaly	Cardiac defect	1
Anencephaly	Embryo arrest	1
Anencephaly	Autism	1
Anencephaly	Others BD, but don't know the type	2
Spina bifida	Spina bifida	3
Spina bifida	Anencephaly	3
Spina bifida	Cerebral palsy	2
Spina bifida	Embryo arrest	2
Spina bifida	Hypospadias	1
Spina bifida	Others BD, but don't know the type	5
Encephalocele	Ear malformation	1

The average interval between NTD pregnancies in our sample was 44.5 ± 32.0 months. The shortest interval was 9 months and the longest was 9 years, with 40% of second pregnancies occurring within 2 years of the first pregnancy and 60% occurring after 2 years or more (Table 5). There were no significant differences in reported dietary frequency except for tea consumption (Supplementary Table 1) between women who had a recurrent NTD and those who did not.

**Table 5:** Interval between two pregnancies with NTDs

Case number	First pregnancy	Second pregnancy	Interval(month)
1	Anencephaly	Encephalocele	46
2	Anencephaly	Spina bifida	108
3	Spina bifida	Spina bifida	19
4	Spina bifida	Spina bifida	42
5	Spina bifida	Spina bifida	9
6	Anencephaly	Spina bifida	18
7	Anencephaly	Spina bifida	71
8	Spina bifida	Anencephaly	31
9	Spina bifida	Anencephaly	79
10	Spina bifida	Anencephaly	22
Mean (SD)			44.5(32.0)

## DISCUSSION

We investigated the recurrence rate of NTDs in a region of historically high prevalence of NTDs in China. Among 578 pregnancies following a previous NTD-affected pregnancy, the total recurrence risk was 1.7%. Among recurrent NTDs, the prevalence among women who did not take folic acid supplements was almost double that of those who take folic acid supplements (2.6% vs 1.5%). The prevalence of NTDs in the study area was 0.32% in 2014, which was approximately ten times higher than in the USA [10] and 2.5 times higher than another northern province Liaoning (0.13%) [18] at same time. The rate of recurrent NTDs was 5 times higher than the prevalence of NTDs in the same area of China.

The recurrence rate in our study was similar to that reported in England, 1.2-1.7% in early 1990s [3, 2], and was much lower than the previously reported recurrence risk of NTDs in China in the early 1990s [13]. This difference in recurrence rate occurred in parallel with a reduction in the overall prevalence of NTDs which may be attributable to the nationwide folic acid supplementation program initiated in 2009. The same trend was found in Liaoning [18]. The program provides free folic acid tablets to all women who have a rural registration and who plan to become pregnant [10, 19, 20].

Clinical trials and the outcome of fortification campaigns show that folic acid supplementation can prevent first occurrence of NTDs and recurrent NTDs. Our study suggests that a lower proportion of women with recurrent NTDs took FA supplements than women who did not have a recurrent NTDs. Among women who had a previous affected pregnancy, less than 50% took FA in the period before their next pregnancy, suggesting that health education initiatives should emphasize the optimal period of supplementation as well as dosage of FA supplement that is protective. As formation of the neural tube occurs during weeks 3-4 of pregnancy, it is too late for women to take folic acid after they know they are pregnant. To obtain optimal blood folate levels to prevent NTDs, it recommended to supplement FA in the periconceptional period (28 days before through 28 days after the last menstrual period) [21]. Moreover, to prevent NTD recurrence, a dose of 4-5 mg FA per day is recommended, as compared with 0.4 mg, based on the findings of the MRC Vitamin study in which the risk of recurrent NTDs was reduced by 72% [6].

Although folic acid supplementation may not be able to prevent all NTDs, it is clear that it has a protective effect. NTDs still arise in regions that have mandated FA fortification of enriched cereal grain products. For example, the rate of NTDs in South Carolina, USA, remained at 0.66 NTDs per 1,000 live births and fetal deaths after 20 years of extensive promotion of periconceptional FA use and 14 years of fortification of cereal grain flour [23]. In contrast to the food fortification approach of the USA and other countries, China implemented a public health campaign to provide FA tablets (0.4 mg per day) to women who may become pregnant. Although the tablets are provided free of charge, the compliance is not satisfactory; our previous study showed that the percentage of FA supplementation before the last menstrual period is around 40% [20]. Folic acid fortification of staple foods could be recommended to further prevent NTDs in China [10].

Our study found that the BMI of women who had a second NTD-affected pregnancy was higher than that of women who did not. It is possible that this contributed to NTD risk, as a higher BMI has been found to be an independent risk factor for NTDs [24, 25]. A population-based case-control study revealed that pre-pregnancy maternal overweight/obese women were more likely than average-weight women to have an infant with spina bifida, heart defects and multiple anomalies [25]. A meta-analysis reported that the odds ratio of NTDs in offspring was 1.04 (95% CI 1.027–1.051) for every 1 kg/m^2^ BMI increase in mothers, and indicated that maternal obesity is an important risk factor for NTDs in offspring [24]. Maternal obesity is a risk factor for adverse pregnancy outcome, suggesting that obesity prevention efforts are needed to increase the number of women who are of healthy weight before pregnancy.

Our study had several strengths. First, it was based on a population-based surveillance system, and the surveillance covered 95.6% of births [7], which ensures quality data. Second, the NTDs were double checked by local doctors and pediatricians from Peking University, which ensures the accuracy of diagnosis. However, several limitations prohibited further analysis. One limitation of the present study was that it may have underestimated the recurrent risk of NTDs because almost half of the participants were not followed up. The second limitation was that it included only five counties, and the results may not be generalizable to the entire province or country. The third limitation was that few risk factors were evaluated, which hindered our ability to explore the risk factors for recurrent NTDs. Future studies should use a larger sample size and collect more information.

In conclusion, the prevalence of recurrent NTDs remains high in the research area, although implementation of the FA supplementation program has been associated with lower overall prevalence of NTDs. Improving the compliance of FA supplementation as a public health campaign could lead to further reduction of the prevalence of NTDs. Some recurrent NTDs arose despite the use of FA supplements, suggesting that further investigations of other NTD risk factors and/or preventive measures are warranted.

## MATERIALS AND METHODS

### Subjects

The study was based on the population-based birth defects surveillance system of five counties (Pingding, Xiyang, Taigu, Zezhou and Shouyang) in northern China [7, 10].

Followed the model of a public health campaign which was to evaluate the effect periconceptional folic acid supplementation to prevent NTDs [26], the surveillance system covers more than 20,000 births in the above five counties each year. Any infant suspected of having a major structural external birth defect by prenatal diagnosis or at delivery is reported by local health care providers. Information of preliminary diagnosis of birth defects and basic demographic information, including birth date, sex, birth order and a detailed clinical description (including measurements where applicable) of the birth defect(s) were collected and a photograph of affected infants was taken within the first 24 h after delivery. Definitive diagnosis, classification and coding of structural birth defects were conducted by a clinical geneticist from Peking University. These data can be used for descriptive epidemiology of birth defects [26]. Details of the surveillance system are available elsewhere [10]. Briefly, all live births (births of 28 or more complete gestational weeks), all stillbirths of at least 20 weeks gestational age, and pregnancy terminations at any gestational age following the prenatal diagnosis of NTDs were included. We identified women who had an NTD-affected pregnancy in the period 2004–2015, and conducted a retrospective survey. We hypothesized that the NTD identified in the surveillance system was the first NTD pregnancy for the woman. If women appeared in the surveillance system a second time or more, we checked the date of the end of the pregnancy to determine which was the first NTD and then further pregnancies were identified as recurrent NTD pregnancies. NTDs included anencephaly (International Classification of Diseases, Tenth Revision, Clinical Modification, ICD-10-CM: 740.0), spina bifida (741.90), and encephalocele (742.0). A structured interview was administrated by a local health care provider, including the birth information of NTDs and recurrent NTDs, and dietary frequency and life style factors. The dietary frequency has been used in our previous study [27] and included the frequency of taking meat, sea food, eggs, milk, fresh vegetables, fresh fruits, legume and local pickled vegetable per week; life style factors included the frequency of tea drinking and passive smoking. An annual work meeting was held for staff who collected and reported data of surveillance system of every county, and the staffs were trained every year. The study protocol was reviewed and approved by the Institutional Review Board of Peking University.

### Statistical analysis

NTDs were classified as “recurrent” if they affected the pregnancy of a mother with a prior NTD-affected pregnancy. NTDs included three major subtypes, namely, anencephaly, spina bifida, and encephalocele.

The prevalence of recurrent NTDs was calculated as the number of recurrent NTDs divided by the number of occurrent NTDs. Data on maternal age, body mass index (BMI) of women before the pregnancy, gestational weeks, education, and the occupation at the time the first NTDs occurred were collected, as were details about NTD types, sex, interval between the two NTDs, FA supplementation, and duration and dosage of FA. Dietary information was compared between affected women and controls. Statistical analyses were conducted using a t-test for comparison of maternal age, BMI, gestational weeks in groups; a Chi-square test were used for comparison of the categorical data, including maternal education, occupation, sex of offspring, FA supplementation information, et al. A two-tailed *P* ≤ 0.05 was considered statistically significant. All statistical analyses were performed using the SPSS package version 20.0 (SPSS Inc.).

## SUPPLEMENTARY MATERIALS FIGURES AND TABLES





## Abbreviations

NTDs: neural tube defects
FA: folic acid
