# Supplementary material for: The recurrence risk of neural tube defects (NTDs) in a population with high prevalence of NTDs in northern China
Source: Oncotarget. 2017 Aug 3;8(42):72577–83. doi: 10.18632/oncotarget.19890 (PMC5641153; doi:10.18632/oncotarget.19890)
Supplement: Supplementary file 1 [file oncotarget-08-72577-s001.pdf]

# The recurrence risk of neural tube defects (NTDs) in a population with high prevalence of NTDs in northern china

## SUPPLEMENTARY MATERIALS

**Supplementary Table 1: Comparison the dietary frequency for the recurrent pregnancy of NTDs and healthy control.**

**See Supplementary File 1**
