# Supplementary material for: The recurrence risk of neural tube defects (NTDs) in a population with high prevalence of NTDs in northern China
Source: Oncotarget. 2017 Aug 3;8(42):72577–83. doi: 10.18632/oncotarget.19890 (PMC5641153; doi:10.18632/oncotarget.19890)
Supplement: Supplementary file 2 [file oncotarget-08-72577-s002.docx]

**Supplementary Table 1: Comparison the dietary frequency for the recurrent pregnancy of NTDs and healthy control**

| **Dietary frequency** | **Recurrent NTDs** | | ***P*^*^** |
| --- | --- | --- | --- |
|  | **Yes** | **No** |  |
| Tea |  |  | <0.001 |
| Never | 9(90.0) | 527(94.8) |  |
| <1 cup/week | 0 | 23(4.1) |  |
| 1-6 cups/week | 0 | 3(0.5) |  |
| 1-2 cups/day | 0 | 2(0.4) |  |
| >2 cups/day | 1(10.0) | 1(0.2) |  |
| Total | 10(100.0) | 556(100.0) |  |
| Passive smoking |  |  | 0.499 |
| Never | 6(60.0) | 214(38.1) |  |
| <1 time/week | 0 | 116(20.6) |  |
| 1-3 times/week | 2(20.0) | 101(18.0) |  |
| 4-6 times/week | 1(10.0) | 65(11.6) |  |
| >6 times/week | 1(10.0) | 66(11.7) |  |
| Total | 10(100.0) | 562(100.0) |  |
| Meat |  |  | 0.212 |
| >6 times/week | 1(10.0) | 22(3.9) |  |
| 4-6 times/week | 2(20.0) | 64(11.4) |  |
| 1-3 times/week | 2(20.0) | 295(52.6) |  |
| <1 time/week | 5(50.0) | 180(32.1) |  |
| Total | 10(100.0) | 561(100.0) |  |
| Sea food |  |  | 0.774 |
| >6 times/week | 0 | 14(2.5) |  |
| 4-6 times/week | 0 | 17(3.1) |  |
| 1-3 times/week | 1(10.0) | 127(22.8) |  |
| <1 time/week | 9(90.0) | 396(71.2) |  |
| Total | 10(100.0) | 556(100.0) |  |
| Eggs |  |  | 0.669 |
| >6 times/week | 1(10.0) | 101(18.0) |  |
| 4-6 times/week | 6(60.0) | 213(38.0) |  |
| 1-3 times/week | 3(30.0) | 213(38.0) |  |
| <1 time/week | 0 | 32(5.7) |  |
| Total | 10(100.0) | 561(100.0) |  |
| Milk |  |  | 0.468 |
| >6 times/week | 1(10.0) | 82(14.7) |  |
| 4-6 times/week | 3(30.0) | 140(25.1) |  |
| 1-3 times/week | 1(10.0) | 179(32.1) |  |
| <1 time/week | 5(50.0) | 154(27.6) |  |
| Total | 10(100.0) | 558(100.0) |  |
| Fresh vegetables |  |  | 0.892 |
| >6 times/week | 4(40.0) | 217(38.9) |  |
| 4-6 times/week | 5(50.0) | 213(38.2) |  |
| 1-3 times/week | 1(10.0) | 120(21.5) |  |
| <1 time/week | 0 | 6(1.1) |  |
| Total | 10(100.0) | 558(100.0) |  |
| Fresh fruits |  |  | 0.733 |
| >6 times/week | 2(20.0) | 216(38.6) |  |
| 4-6 times/week | 4(40.0) | 188(33.6) |  |
| 1-3 times/week | 4(40.0) | 143(25.6) |  |
| <1 time/week | 0 | 10(1.8) |  |
| Total | 10(100.0) | 559 (100.0) |  |
| Legume |  |  | 0.913 |
| >6 times/week | 1(10.0) | 69(12.3) |  |
| 4-6 times/week | 1(10.0) | 114(20.4) |  |
| 1-3 times/week | 6(60.0) | 287 (51.3) |  |
| <1 time/week | 2(20.0) | 84(15.0) |  |
| Total | 10(100.0) | 560(100.0) |  |
| Local pickled vegetable |  |  | 0.627 |
| >6 times/week | 1(10.0) | 23(4.1) |  |
| 4-6 times/week | 1(10.0) | 18(3.2) |  |
| 1-3 times/week | 2(20.0) | 169(30.2) |  |
| <1 time/week | 6(60.0) | 345(61.6) |  |
| Total | 10(100.0) | 560(100.0) |  |

^*^Compared between recurrent NTDs and the second pregnancy without NTDs
